# Angiotensin II Stimulates the Proliferation and Migration of Lymphatic Endothelial Cells Through Angiotensin Type 1 Receptors

**DOI:** 10.3389/fphys.2020.560170

**Published:** 2020-09-08

**Authors:** Qiu-Yue Lin, Jie Bai, Jin-Qiu Liu, Hui-Hua Li

**Affiliations:** Department of Cardiology, Institute of Cardiovascular Diseases, The First Affiliated Hospital of Dalian Medical University, Dalian, China

**Keywords:** angiotensin II, lymphatic endothelial cells, proliferation, migration, microarray, time-series gene expression profiling

## Abstract

**Background/Aim:**

The proliferation and migration of lymphatic endothelial cells (LECs) is essential for lymphatic vessel growth (also known as lymphangiogenesis), which plays a crucial role in regulating the tissue fluid balance and immune cell trafficking under physiological and pathological conditions. Several growth factors, such as VEGF-C, can stimulate lymphangiogenesis. However, the effects of angiotensin II (Ang II) on the proliferation and migration of mouse LECs and the underlying potential mechanisms remain unknown.

**Methods:**

Wild-type mice were infused with Ang II (1,000 ng/kg/min) for 1–2 weeks. Murine LECs were stimulated with Ang II (500 nM) or saline for 12–48 h. Cell proliferation was determined with 5-bromo-2-deoxyuridine (BrdU) incorporation assays, while cell migration was assessed by scratch wound healing and transwell chamber assays. The gene expression profiles were obtained by time series microarray and real-time PCR analyses.

**Results:**

Ang II treatment significantly induced lymphangiogenesis in the hearts of mice and the proliferation and migration of cultured LECs in a time-dependent manner. This effect was completely blocked by losartan, an angiotensin II type 1 receptor (AT1R) antagonist. The microarray results identified 1,385 differentially expressed genes (DEGs) at one or more time points in the Ang II-treated cells compared with the control saline-treated cells. These DEGs were primarily involved in biological processes and pathways, including sensory perception of smell, the G protein coupled receptor signaling pathway, cell adhesion, olfactory transduction, Jak-STAT, alcoholism, RIG-I-like receptor and ECM-receptor interaction. Furthermore, these DEGs were classified into 16 clusters, 7 of which (Nos. 13, 2, 8, 15, 7, 3, and 12, containing 586 genes) were statistically significant. Importantly, the Ang II-induced alterations the expression of lymphangiogenesis-related genes were reversed by losartan.

**Conclusion:**

The results of the present indicate that Ang II can directly regulate the proliferation and migration of LECs through AT1R *in vivo* and *in vitro*, which may provide new potential treatments for Ang II-induced hypertension and cardiac remodeling.

## Introduction

The lymphatic system comprises lymphatic capillaries, pre-collecting vessels and collecting vessels, all of which are composed of a single layer of lymphatic endothelial cells (LECs) (Hu et al., 2019). Several LEC markers, such as lymphatic vessel hyaluronan receptor-1 (LYVE-1), Prox1, Foxc2, CCL21, and vascular endothelial growth factor (VEGF) receptor-3 (VEGFR-3) are all highly expressed in most lymphatic vessels during development. After maturation, lymphatic capillaries continue to express high levels of LYVE-1, Prox1, VEGFR-3, and low level of Podoplanin (Yang et al., 2012). The lymphatic system is crucial for the maintenance of interstitial fluid balance, immunocyte surveillance, and the reabsorption of proteins and lipids from the intestine (Hu et al., 2019). However, lymphatic dysfunction, either due to gene mutations or secondary to damage to the lymphatic vessels, may lead to lymphedema, chylothorax, inflammation, and tumor metastasis (Aspelund et al., 2016; Hu et al., 2019; Xiao et al., 2019). Interestingly, there are several reports indicating a relationship between cardiac lymphatic dysfunction, edema, and contractile dysfunction in animals with myocardial infarction (MI) or ischemia/reperfusion (I/R) injury (Klotz et al., 2015; Henri et al., 2016; Shimizu et al., 2018; Vuorio et al., 2018). Therefore, it is important to elucidate the regulatory mechanisms underlying lymphatic vessel growth (also known as lymphangiogenesis).

Numerous growth factors, including angiopoietins and VEGF, play crucial roles in lymphangiogenesis by stimulating the proliferation and migration of LECs, with VEGF-C and its receptor VEGFR-3 being the central signaling molecules in this process (Norrmén et al., 2011). Accumulating evidence has demonstrated that monocytes are the source of VEGF-C in injured kidneys (Lee et al., 2013). Angiotensin II (Ang II), a major hormone effector of the renin-angiotensin-system (RAS), plays a crucial role in inflammation and oxidative stress, which lead to increased hypertension and cardiac remodeling (Wang et al., 2018). The results of previous studies have indicated that Ang II can induce the secretion of VEGF-C by activated immunocytes *in vitro*, which further enhances renal lymphatic vessel density and prevents hypertension in mice (Balasubbramanian et al., 2020). Conversely, the administration of angiotensin II type 1 receptor blockers (ARBs), such as telmisartan, can partially inhibit TNF-α-induced VEGF-C production in human proximal renal tubular epithelial cells (Kimura et al., 2014). Moreover, the administration of angiotensin-converting enzyme inhibitors (ACEIs) or ARBs to mice can inhibit lymphangiogenesis in gastric cancer, thereby suppressing tumor growth (Wang et al., 2008). However, whether Ang II stimulates lymphangiogenesis directly or indirectly by inducing VEGF-C and the associated underlying mechanism remains to be elucidated.

In the present study, we evaluated the effect of Ang II on lymphangiogenesis in the mouse hearts and the gene expression profiles in cultured primary mouse LECs using time-series microarrays and attempted to elucidate the possible mechanism by which Ang II regulates LEC proliferation and migration, which are crucial for lymphangiogenesis.

## Materials and Methods

### Animals and Treatment

C57BL/6J male mice (10 weeks-old, *n* = 6/group) were subcutaneously infused with Ang II (1,000 ng/kg/min; Sigma-Aldrich, St. Louis, MO, United States) via implanted osmotic minipumps (Model 1007D, Alzet, Cupertino, CA, United States) for 1–2 weeks as previously described (Wang et al., 2019). The mice were intravenously injected with losartan (10 mg/kg; HY-17512, MedChemExpress, United States) beginning at 1 day before Ang II infusion and treated concurrently for 1–2 weeks. Blood pressure was measured by the tail-cuff method before starting treatment and every 2 days after Ang II infusion as previously described (Wang et al., 2016, 2019). Mice were intraperitoneally anesthetized with an overdose of tribromoethanol (0.4 mg/g). All procedures were approved by the Animal Care and Use Committee of Dalian Medical University, and conformed to the Guide for the Care and Use of Laboratory Animals published by the U.S. NIH.

### Cell Culture and Treatment

Mouse lymphatic endothelial cells (mLECs) were purchased from Cellbio Company (CBR-131164), and grown in DMEM basic medium supplemented with 10% fetal bovine serum (FBS) and 1% penicillin-streptomycin under an atmosphere of 5% CO_2_ at 37°C as previously described (Balasubbramanian et al., 2020). For *in vitro* cell assays and gene expression profile analysis, mLECs were treated with Ang II (0.5 nM; Sigma-Aldrich, St. Louis, MO, United States) at a dose of 500 nM in culture medium for different time points (12, 24, and 48 h). mLECs were also cotreated with losartan (10 μM; HY-17512, MedChemExpress, United States) and Ang II (0.5 nM; Sigma-Aldrich, St. Louis, MO, United States) in culture medium for 24 h.

### Immunostaining

Immunostaining was performed as previously described (Wang et al., 2018). mLECs were identified by immunostaining with anti-LYVE-1 (NBP1-43411, Novus) and anti-VEGFR-3 (ab27278, Abcam) antibodies, as both of which are the selective markers for lymphatic endothelium. Briefly, cells were washed with PBS three times, fixed with 4% paraformaldehyde for 30 min, permeated with 0.1% Triton X-100 for 15 min, and then blocked with 5% bovine serum albumin. Cells or heart frozen sections were incubated with primary antibodies against LYVE-1 and VEGFR-3 (diluted 1:100) overnight at 4°C and then incubated with a red fluorescent secondary antibody (diluted 1:200; A0453, Beyotime) for 2 h at room temperature. The cell nuclei were counterstained with 4′,6-diamidino-2-phenylindole (DAPI) and are shown in blue. The images were analyzed using a Nikon microscope (Tokyo, Japan).

### BrdU Incorporation Assay

Lymphatic endothelial cells proliferation was assessed using 5-bromo-2-deoxyuridine (BrdU; KeyGEN company, KGA337-100) according to the manufacturer’s instructions. Briefly, cells were grown on glass coverslips and cultured in serum-free medium with Ang II (500 nM) for 12–48 h. Subsequently, the cells were incubated with 30 nmol/L BrdU for 4 h in DMEM and then fixed with 4% formaldehyde, 2 mg/mL glycine solution was used to neutralize residual formaldehyde and then counterstained with DAPI. The EdU was detected by Click-iT reaction mixture and fluorescence images were obtained using a fluorescence microscope (Nikon, Tokyo, Japan) at 100 × magnification. The BrdU^+^ cells were counted using ImageJ as previously described (Thompson and Romano, 2016; Xu et al., 2019).

### Scratch Wound Healing Assay

A scratch wound healing assay was performed as previously described (Xu et al., 2019). The LECs were pre-grown to confluence in a monolayer in 6-well plates. Then, a linear scratch approximately 1-mm wide was generated in the monolayer of cells on the plate surface with a 200-μl sterile pipette tip held perpendicularly to the plate. Then, the cells were re-cultured in serum-free DMEM with Ang II (500 nM) for 12–48 h, after which the relative area of the scratch for different cell groups was analyzed using ImageJ.

### Transwell Chamber Assay

The transwell migration assay was performed as previously described (Wang et al., 2018). Briefly, 5 × 10^4^ mLECs were suspended in 200 μl DMEM and added to the upper chambers of transwell inserts (8 μm pore, Corning, New York, NY, United States) in a 24-well plate. Then, conditioned medium collected from Ang II-pretreated mLECs after starvation for 4 h was added to the lower chambers of the plate, and the cells were cultured for 12–48 h. Subsequently, the migrated cells on the lower surface of the inserts were fixed with 4% formaldehyde and stained with DAPI, and the migrated cells in six randomly selected fields were counted using ImageJ.

### Microarray and Bioinformatics Analyses

The mLECs were pretreated with Ang II for 12 or 24 h, and total RNA was extracted from the cells using TRIzol (Invitrogen, United States) according to the manufacturer’s instructions. RNA was biotin-labeled after evaluating RNA quality and integrity and placed on a Gene Chip Hybridization Oven 645 instrument. A Gene Chip Fluidics Station 450 was used to wash and stain the cells of the chip, and a Gene Chip 3000 7G scanner was used to measure the fluorescence signal. The CEL files of each signal value of the gene probe were generated using GCOS. mRNA expression profiling was performed using a mouse Genome 430 2.0 Array (Affymetrix Gene Chip, United States) according to the manufacturer’s instructions. The Affymetrix Gene Chip used in the current study contains 221,900 probes, and an average of 10 probes were used to assess each gene of interest after removing the quality control probe. The error rate-corrected data were analyzed with Transcriptome Analysis Console (TAC4.0) and normalized using the Robust Multichip Analysis (RMA) algorithm. The fold change in gene expression of Ang II-treated cells for every time point was normalized to the control group. Hierarchical clustering (visualized with a heatmap), Gene Ontology (GO), Kyoto Encyclopedia of Genes and Genomes (KEGG) pathway, global signal transduction network, co-expression network and gene expression trend bioinformatics analyses were performed as previously described (Wu et al., 2019). Statistical analyses of microarray and bioinformatics data were performed with Fisher’s exact test and Duncan’s multiple range test to calculate the *P*-value and FDR. *P* < 0.01 was considered a significant difference.

### Quantitative Real-Time PCR Analysis

Total RNA was extracted from cells or fresh heart tissues using TRIzol reagent (Invitrogen, United States) according to the manufacturer’s protocols. First-strand cDNA was produced from 1 μg of total RNA from each sample using PrimeScript RT reagent kit (Takara, RR047A) according to the manufacturer’s instruction. The mRNA levels of genes (LYVE-1, Prox1, VEGF-C, VEGFR-3, AT1R, Ace, Dusp10, Hes1, Hist1H2Ak, Psmc4, Klf9, Olfr77, Olfr1195, Kdr, Jun, Sparc, Ets1, Stc1, and GAPDH) were analyzed using SYBR Green Premix (ACCURATE BIOTECHNOLOGY (HUNAN) Co., Ltd., AG11701) on an Applied Biosystems 7500 Fast thermocycler (ABI, United States). The primers used in the present study are listed in Table 2. The target gene expression level was normalized to the expression of internal reference gene glyceraldehyde-3-phosphate dehydrogenase (GAPDH).

### Statistical Analysis

The results are presented as the means ± standard deviation (SD). For cell proliferation, migration and quantitative RT-PCR analyses, the differences among groups were determined using one-way ANOVA in GraphPad Prism 7.0. Differences were considered to be significant at *P* < 0.05.

## Results

### Ang II Infusion Increases Cardiac Lymphangiogenesis via AT1R *in vivo*

**FIGURE 1:**
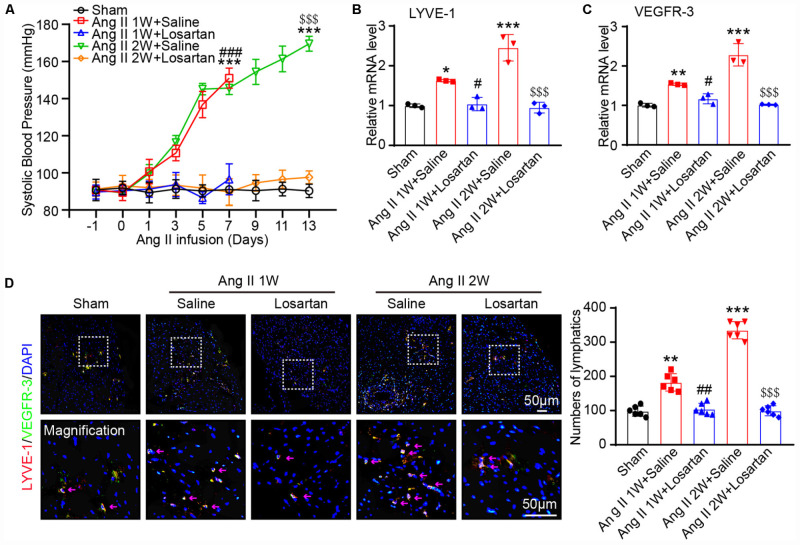
Ang II infusion increases cardiac lymphangiogenesis via AT1R *in vivo*. **(A)** Wild-type mice were subcutaneously infused with Ang II (1,000 ng/kg/min) with or without losartan (10 mg/kg) for 1 or 2 weeks, and the systolic blood pressure was measured by the tail-cuff method (*n* = 6). **(B)** Cardiac mRNA expression level of LYVE-1 was measured by qPCR analysis (*n* = 3). **(C)** Cardiac mRNA expression level of VEGFR-3 was measured by qPCR analysis (*n* = 3). **(D)** Immunofluorescence staining of hearts with anti-LYVE-1 (Red) and anti- VEGFR-3 (Green) antibodies and DAPI (Blue) (Left, *n* = 6), and the quantification of the LYVE-1^+^ and Prox1^+^ lymphatic vessels in the hearts (Right, *n* = 6). The results are expressed as the means ± SD, and n represents the number of independent experiments. *P < 0.05, **P < 0.01, and ***P < 0.001 versus Sham group; ^#^*P* < 0.05, ^##^*P* < 0.01, and ^###^*P* < 0.001 versus Ang II 1W + Saline group; ^$$$^*P* < 0.001 versus Ang II 2W + Saline group.

To investigate the role of Ang II in the regulation of lymphangiogenesis in the heart, wild-type mice were subcutaneously infused with Ang II (1,000 ng/kg/min) in the presence or absence of losartan (10 mg/kg) for 1 or 2 weeks. We observed that the Ang II infusion-induced elevation of systolic blood pressure (SBP) was completely blocked by losartan at 1 and 2 weeks (Figure 1A). Moreover, Ang II infusion significantly upregulated the mRNA expression levels of both LYVE-1 and VEGFR-3, markers of lymphangiogenesis, in the heart tissues compared with that observed in the sham control, whereas this increase was fully inhibited by losartan administration (Figures 1B,C). In addition, immunostaining results confirmed that the Ang II-stimulated increased LYVE-1^+^ (red) and VEGFR-3^+^ (green) lymphatic endothelial cells in the hearts at 1 week and multicellar vessels at 2 weeks that was abrogated in losartan-treated hearts (Figure 1D). Thus, these results indicate that Ang II promotes cardiac lymphangiogenesis through AT1R.

### Ang II Promotes the Proliferation and Migration of LECs *in vitro*

**FIGURE 2:**
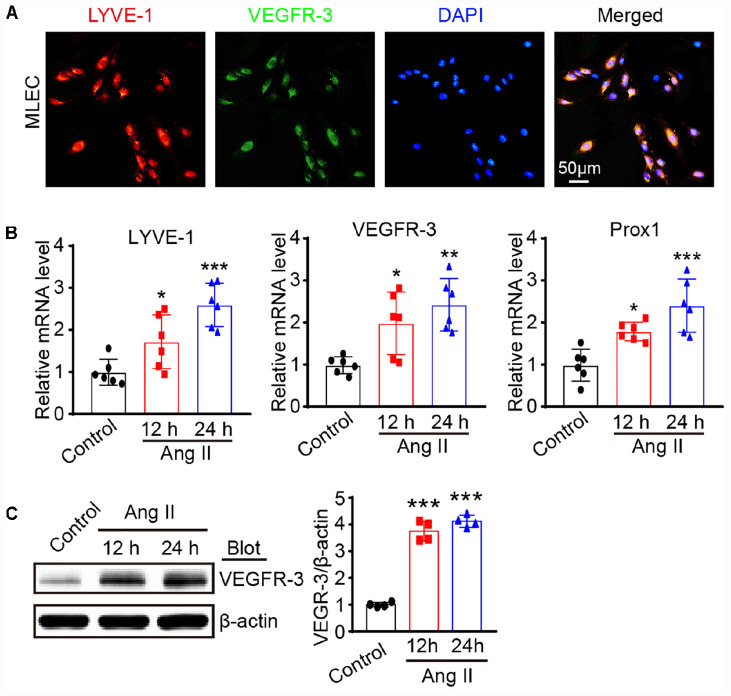
Ang II treatment promotes lymphatic marker expression of LECs *in vitro*. **(A)** Immunofluorescence staining of LECs with anti-LYVE-1 and anti-VEGFR-3 antibodies**. (B)** LECs were treated with Ang II (500 nM) for 12 and 24 h, the mRNA levels of LYVE-1, VEGFR-3 and Prox1 were detected by qPCR, and the data were normalized using the reference gene GAPDH (*n* = 6). **(C)** The protein expression level of VEGFR-3 was measured by immunoblotting and normalized using β-actin (*n* = 4). The results are expressed as the means ± SD, and n represents the number of independent experiments. *P < 0.05, **P < 0.01, and ***P < 0.001 versus Control.

To determine the effect of Ang II on the proliferation and migration of LECs, we first analyzed LECs by immunofluorescence staining with antibodies against to LYVE-1 and VEGFR-3, respectively. The results presented in Figure 2A showed that both LYVE-1 (red) and VEGFR-3 (green) were expressed and colocalized on the surface of the LECs. Moreover, qPCR analysis revealed that the mRNA levels of lymphatic markers such as LYVE-1, VEGFR-3 and Prox1 as well as VEGRFR-3 protein levels were expressed in LECs and significantly upregulated after Ang II treatment compared with that observed in the saline control group (Figures 2B,C), indicating these cells have the characteristics of LECs.

**FIGURE 3:**
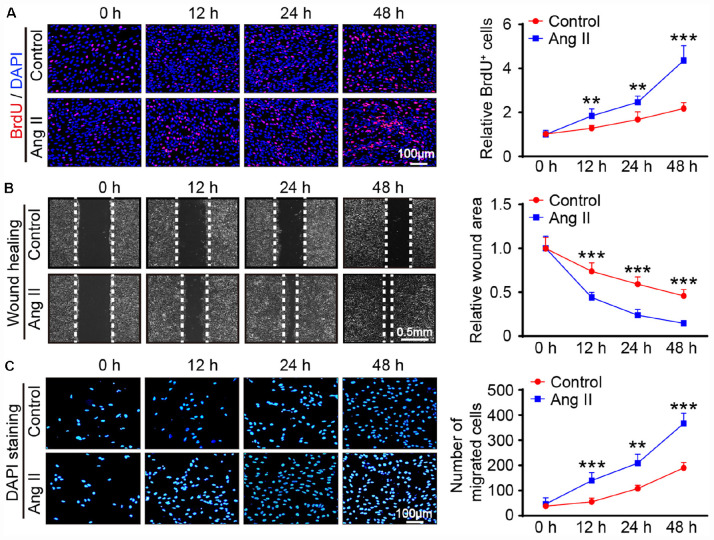
Ang II treatment promotes the proliferation and migration of LECs *in vitro*. **(A)** LECs were treated with Ang II (500 nM) for 12, 24, and 48 h, and cell proliferation capability was measured by a BrdU assay (Left) and the relative quantitation of BrdU^+^ cells (Right, *n* = 3). Scale bar = 100 μm. **(B)** Cell migration was examined by scratch wound healing assay (Left) and the relative quantitation of wound area (Right, *n* = 3). Scale bar = 100 μm. **(C)** Cell migration was detected by a Transwell assay (Left) and the relative quantitation of DAPI^+^ cells (Right, *n* = 3). Scale bar = 100 μm. The results are expressed as the means ± SD, and n represents the number of independent experiments. **P < 0.01, and ***P < 0.001 versus Control.

We then evaluated the effect of Ang II on cell growth. BrdU incorporation assay results showed that Ang II increased the proliferation of mLECs in a time-dependent manner (Figure 3A). The migration capability of LECs was also examined by scratch wound healing and transwell chamber assays. Compared with that observed in the control treatment, the Ang II treatment reduced the wound area in a time-dependent manner (Figure 3B) and increased the number of migrated cells (Figure 3C).

### Ang II Induces LECs Proliferation and Migration via AT1R *in vitro*

**FIGURE 4:**
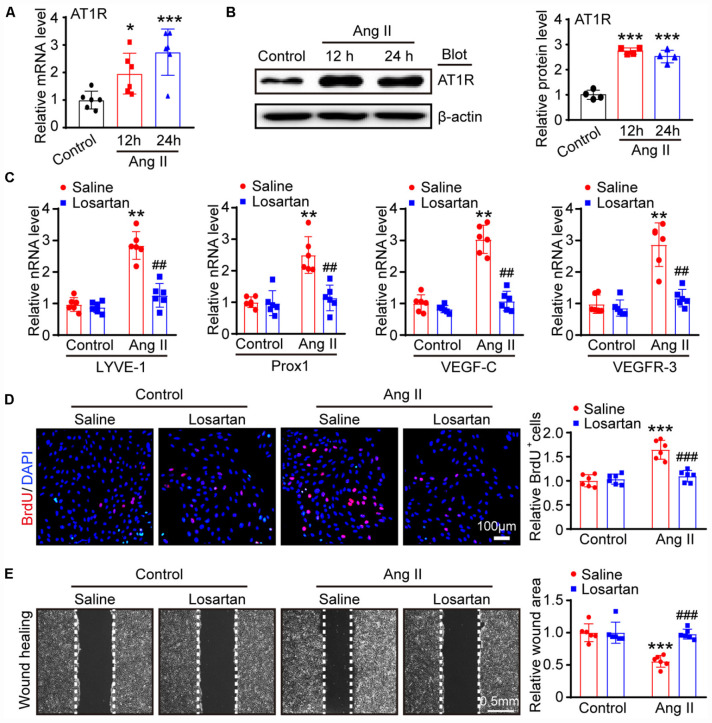
Ang II induces LECs proliferation and migration via AT1R *in vitro*. **(A)** LECs were treated with Ang II (500 nM) for 12 and 24 h, and the AT1R mRNA level was measured by qPCR (*n* = 6). **(B)** The AT1R protein expression level was measured by immunoblotting and normalized using β-actin (*n* = 4). **(C)** LECs pretreated with losartan (10 μM) and then stimulated with Ang II for an additional 24 h, the mRNA expression levels of lymphatic markers for LYVE-1, Prox1, VEGF-C and VEGFR-3 were measured by qPCR (*n* = 6). **(D)** Cell proliferation capability was measured by a BrdU assay (Left) and the relative quantitation of BrdU^+^ cells (Right, *n* = 6). Scale bar = 100 μm. **(E)** Cell migration was examined by scratch wound healing assay (Left) and the relative quantitation of wound area (Right, *n* = 6). Scale bar = 100 μm. The results are expressed as the means ± SD, and n represents the number of independent experiments. *P < 0.05, **P < 0.01, and ***P < 0.001 versus Control + Saline; ^##^*P* < 0.01, and ^###^*P* < 0.001 versus Ang II + Saline.

To confirm whether AT1R mediates the Ang II-induced proliferation and migration of LECs, we first examined the expression of AT1R in LECs. qPCR and immunoblotting results showed that AT1R expression at the mRNA and protein levels was in LECs was markedly upregulated after Ang II treatment (Figures 4A,B), indicating that AT1R is expressed in mLECs. To determine whether Ang II directly acts via AT1R, LECs were pretreated with losartan (10 μM) and then stimulated with Ang II for an additional 24 h. qPCR results showed that the mRNA levels of lymphangiogenesis-related genes, such as LYVE-1, Prox1, VEGF-C and VEGFR-3 were significantly increased in the Ang II-treated LECs compared with that observed in the saline treatment group, whereas, these increases were fully abolished by losartan treatment (Figure 4C). Moreover, BrdU incorporation and scratch wound healing assay results further confirmed that Ang II treatment markedly promoted the proliferation and migration of LECs compared with that observed by the control treatment (Figures 4D,E). Conversely, this Ang II-mediated effect was completely attenuated by losartan treatment (Figures 4D,E). Losartan had no significant effect on LECs in the control treatment group (Figures 4C,E). Taken together, these results demonstrate that Ang II directly promotes the proliferation and migration of LECs via AT1R *in vitro*.

### Differentially Expressed Genes and GO and KEGG Pathway Analyses in Ang II-Treated LECs

**FIGURE 5:**
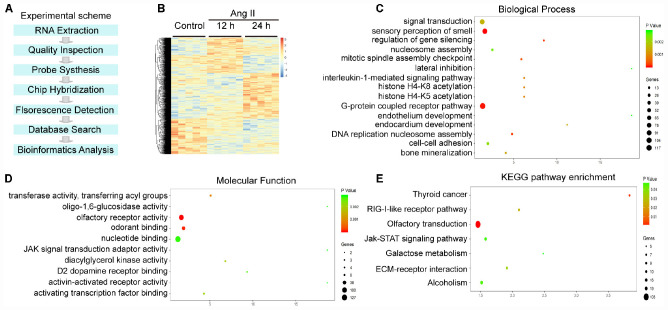
Analysis of hierarchical clustering, GO analysis and KEGG pathway analysis in Ang II-treated LECs. **(A)** Experimental scheme for the obtaining of gene expression profiles. **(B)** Heat map of 1,385 differentially expressed genes from the microarrays between Ang II-treated LECs and saline control at 12 and 24 h (*n* = 5 per group). Orange and blue indicate upregulation and downregulation of mRNA expression, respectively. **(C)** BP analysis of the differentially expressed genes. **(D)** GO analysis of the molecular function of the differentially expressed genes. **(E)** Analysis of KEGG pathways for the differentially expressed genes. The size and color of each bubble indicate the number of differentially expressed genes and the statistical *P* value of each enrichment.

To identify the gene expression profiles during the Ang II-induced proliferation and migration of LECs, we performed a time-series microarray analysis of LECs 12 and 24 h after Ang II treatment (*n* = 5 per time point). The experimental scheme is shown in Figure 5A. The microarray results identified 1,385 genes that were differentially expressed in the Ang II-treated LECs for at least at one time point when compared with the expression in the control cells (*P* < 0.05 and FDR < 0.05), with the data displayed in a heatmap (Figure 5B). Among these genes, 712 and 717 were upregulated at 12 or 24 h, respectively. Conversely, 673 and 668 genes were downregulated at 12 or 24 h, respectively (Figure 5B).

To identify the biologically significant genes in LECs induced by Ang II treatment, we performed GO enrichment analysis of altered genes categorized by biological process (BP). The differentially expressed genes were primarily involved in sensory perception of smell, G protein coupled receptor pathway, regulation of gene silencing, DNA replication-dependent nucleosome assembly, cell adhesion and an interleukin-1-mediated signaling pathway (Figure 5C). Moreover, most molecular functions included olfactory receptor activity, odorant and nuclear binding, transferase activity and transferring acyl groups (Figure 5D).

We then performed a KEGG pathway analysis of the genes showing significantly altered expression. The results indicated that 8 pathways were significantly altered in the Ang II-treated LECs (*P* < 0.05), which included olfactory transduction, thyroid cancer, RIG-I-like receptor signaling pathway, ECM-receptor interaction, alcoholism, the Jak-STAT signaling pathway and galactose metabolism (Figure 5E). Overall, these results indicate that these GO terms and pathways may play crucial roles in Ang II-induced lymphangiogenesis.

### Analysis of Gene Expression Clusters of Ang II-Treated LECs

**FIGURE 6:**
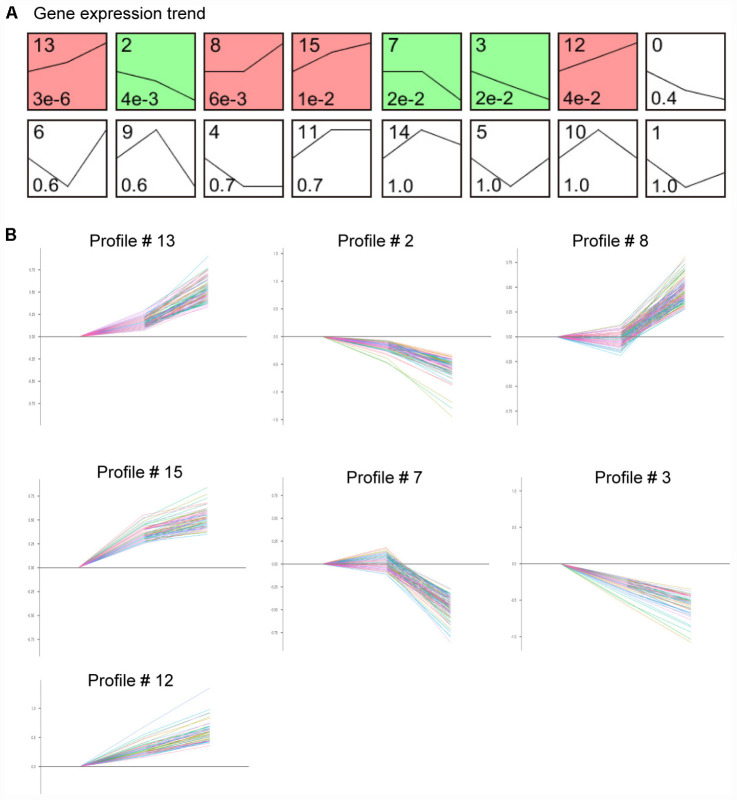
Time-series analysis of differentially expressed genes in each pattern in Ang II-treated LECs. **(A)** Gene expression trend analysis of mRNA expression trends for 1,385 differentially expressed genes. **(B)** Seven gene expression patterns (profile # 13, 2, 8, 15, 7, 3, and 12) were statistically significant (*n* = 5 per group, *P* < 0.05). The *X*-axis indicates different time points of Ang II treatment, and the *Y*-axis indicates the average logarithm of the expression change value.

We next assessed the temporal expression patterns of the 1,385 differently expressed genes (DEGs) in the Ang II-treated LECs at 12 and 24 h. The expression profiles of the DEGs were classified into 16 categories according to their functions and expression trends, of which 7 profiles (Nos. 13, 2, 8, 15, 7, 3, and 12) containing 586 genes were statistically significant (Figure 6A). Moreover, the differentially expressed genes in profile Nos. 13, 8, 15 and 12 progressively increased in time-dependent patterns after Ang II treatment, whereas that of genes in profile Nos. 2, 7, and 3 gradually decreased at 12 and 24 h (Figure 6B).

### Validation of Gene Expression in Ang II-Treated LECs by qPCR Analysis

**FIGURE 7:**
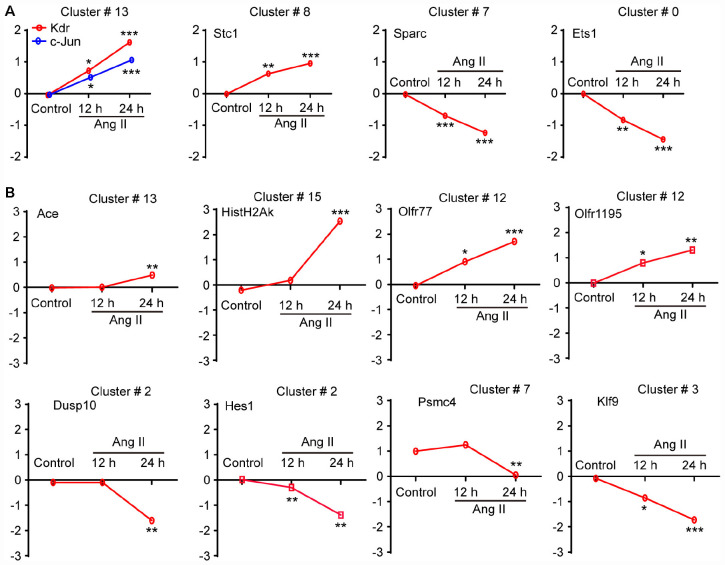
Validation of the gene expression profiles from the microarray by qPCR analysis. **(A)** The mRNA expression level analyses of Kdr, c-Jun, Stc1, Sparc and Ets1, which have been reported to be associated with LEC migration and proliferation or lymphangiogenesis (*n* = 6). **(B)** The mRNA expression levels of 8 selected genes contained in the seven differential expression profiles were verified by qPCR analysis, including No. 13 (Ace), 15 (Hist1H2Ak), 12 (Olfr77 and Olfr1195), 2 (Dusp10 and Hes1), 7 (Psmc4) and 3 (Klf9), which were normalized using GAPDH as an internal control (*n* = 6). The *X*-axis indicates different time points of Ang II treatment, and the *Y*-axis indicates the average logarithm of the expression change value. The results are expressed as the means ± SD, and n represents the number of independent experiments. *P < 0.05, **P < 0.01, and ***P < 0.001 versus Control.

To validate the findings obtained from the microarray (Table 1), we first analyzed the mRNA levels of Kdr, c-Jun (cluster 13), Stc1 (cluster 8), Sparc (cluster 7), and Ets1 (cluster 0), which have been reported to be associated with LECs migration and proliferation or lymphangiogenesis (Wissmann and Detmar, 2006; Gutierrez et al., 2013; Vogrin et al., 2019). The qPCR results revealed that Ang II treatment upregulated the mRNA levels of Kdr, c-Jun and Stc1 but reduced the mRNA levels of Sparc and Ets1 in LECs in a time-dependent manner compared with control (Figure 7A). Moreover, we also examined 9 DEGs selected from 7 statistically significant clusters and observed that Ang II treatment significantly upregulated the mRNA levels of Ace (Cluster 13), Hist1H2Ak (cluster 15), and Olfr77 and Olfr1195 (cluster 12) in LECs in a time-dependent manner (Figure 7B). Conversely, the mRNA levels of Dusp10 and Hes1 (cluster 2), Psmc4 (cluster 7), and Klf9 (cluster 3) were markedly downregulated with increasing time in the Ang II-treated LECs (Figure 7B). These results were in agreement with the data from the microarray analysis (Table 1).

**TABLE 1:** Analysis of differently expressed genes in Ang II-treated LECs by microarray.

Gene	Gene description	FC	*P-*value	Profile
symbol				
Kdr	Kinase insert domain protein receptor	1.55	0.0206	13
c-Jun	Jun proto-oncogene	1.71	0.0062	13
Stc1	Stanniocalcin 1	1.33	0.0429	8
Sparc	Secreted acidic cysteine rich glycoprotein	–1.29	0.0326	7
Ets1	E26 avian leukemia oncogene 1, 5 domains	–1.49	0.0064	0
Ace	Angiotensin I converting enzyme 1	1.40	0.0411	13
Hist1H2AK	Histone cluster 1, H2ak	1.79	0.0014	15
Olfr77	Olfactory receptor 77	2.55	0.0012	12
Olfr1195	Olfactory receptor 1195	1.97	0.0014	12
DUSP10	Dual specificity phosphatase 10	–2.73	0.0000	2
Hes1	Hairy and enhancer of split 1 (Drosophila)	–2.64	0.0000	2
Psmc4	Proteasome 26S subunit, ATPase, 4	–1.83	0.0013	7
Klf9	Kruppel-like factor 9	–1.92	0.0063	3

**TABLE 2:** Primer sequences.

Gene	Forward primer (5′–3′)	Reverse primer (5′–3′)
LYVE-1	GCCAACGAGGCCTGTAAGAT	TCCAACCCATCCATAGCTGC
Prox1	CGTGAAGTTCAACAGAT GCATTA	CAAAGTCATTTGCTTTGTT GTAGTG
VEGFR-3	CCGCAAGTGCATTCACAGAG	TCGGACATAGTCGGGGTCTT
AT1R	ATGCTTGGGGCAACTTCACTA	CGGTGCATGTGGTAGACGAG
VEGF-C	TGTGCTTCTTGTCTCTGGCG	CCTTCAAAAGCCTTG ACCTCG
Kdr	TTTGGCAAATACAACCC TTCAGA	GCTCCAGTATCATTTC CAACCA
c-Jun	TTCCTCCAGTCCGAGAGCG	TGAGAAGGTCCGAGT TCTTGG
Stc1	CTCCAAAACTCAGCAGT GATTCT	GAGGCAGCGAACCACTTCA
Sparc	TGGGAGAATTTGAGG ACGGTG	GAGTCGAAGGTCTTG TTGTCAT
Ets1	GATCTCAAGCCGAC TCTCACC	GACGTGGGTTTCTGTCCACT
Ace	AGCCCAAGTGTTGT TGAACGA	TGGATACCTCCGTGCTTTTCT
Hist1H2Ak	ACCACTTACTGAGCAGGCTT	CGCTCCGAGTAGTTGCCTTT
Olfr77	ACCCTCGAATCTGTGGCCT	AAGCTCAGTGAGTTCA CAAAAGA
Olfr1195	GTGAGTTTCCTTATGCT CATGGT	CTTGTCCATAGGGAA GGTGGT
Dusp10	CATCTCCTTTAGACGA CAGGGT	GGAGCGTGGCTACCACTAC
Hes1	TCAACACGACACCGG ACAAAC	ATGCCGGGAGCTATCTTTCTT
Psmc4	CAGCACTGTCCGTGTCTCG	GATGACCAAGGGGATGCTCT
Klf9	GCCGCCTACATGGACTTCG	GGTCACCGTGTTCCTTGGT
GAPDH	GGTTGTCTCCTGCGACTTCA	GGTGGTCCAGGGTTT CTTACTC

### Ang II Regulates Lymphatic Gene Expression Through AT1 Receptor

**FIGURE 8:**
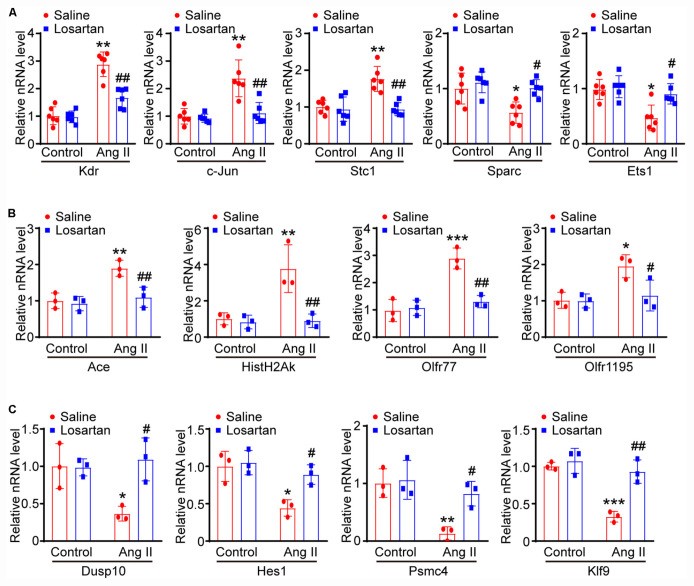
Ang II regulates lymphatic gene expression through AT1 receptor. **(A)** LECs were pretreated with control or losartan and then stimulated with saline or Ang II for 24 h, the lymphatic-related genes of Kdr, c-Jun, Sparc, Ets1 and Stc1 were analyzed by qPCR and normalized by GAPDH (*n* = 6). **(B)** qPCR analyses of Ace, Hist1H2Ak, Olfr77 and Olfr1195 gene expression levels with GAPDH internal control gene (*n* = 6). **(C)** qPCR analyses of Dusp10, Hes1, Psmc4, and Klf9 mRNA levels with GAPDH internal control gene (*n* = 6). The results are expressed as the means ± SD, and n represents the number of independent experiments. *P < 0.05, **P < 0.01, and ***P < 0.001 versus Control. ^#^*P* < 0.05, ^##^*P* < 0.01, and ^###^*P* < 0.001 versus Ang II + Saline.

To further elucidate the signaling pathway whereby Ang II-induced changes of lymphangiogenesis-related gene expression, LECs were pretreated with control or losartan and then stimulated with saline or Ang II for 24 h. The qPCR results demonstrated that Ang II-induced increased Kdr, c-Jun, Stc1, Ace, Hist1H2Ak, Olfr77, and Olfr1195 mRNA levels and that decreased of Sparc and Ets1 Dusp10, Hes1, Psmc4, and Klf9 mRNA levels were markedly reversed in losartan-treated LECs (Figures 8A,B), indicating that the effect of Ang II on the gene expression of LECs is AT1R dependent.

### Gene Co-expression Network Analysis

**FIGURE 9:**
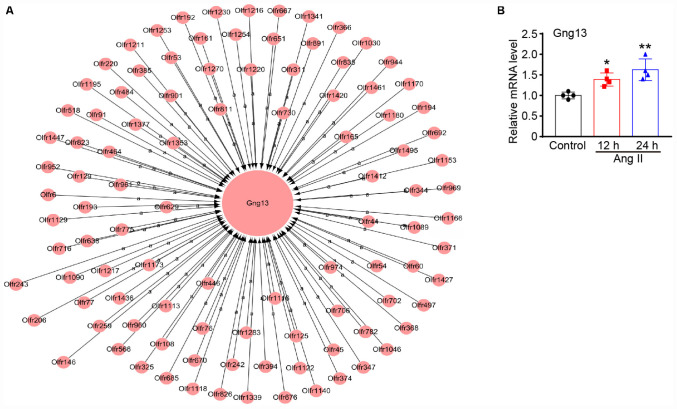
**(A)** Analysis of the gene co-expression network. A total of 104 genes selected from 7 significant profiles were further subjected to a gene co-expression network with the k-core algorithm. Gng13 was localized in the core of the network based on the degree, *k*-core value, and betweenness centrality. The cycle node shows genes, and the edge between two nodes represents the interaction between genes. **(B)** The mRNA level of gng13 was verified by qPCR analysis in Ang II-treated LECs (*n* = 4 per group). The results are expressed as the mean ± SD, and n represents the number of independent experiments. *P < 0.05 and **P < 0.01 versus Control.

To assess which gene plays a crucial role in Ang II-induced lymphangiogenesis, we further performed a gene co-expression network in 7 significant profiles. Based on the degree, *k*-core value, and betweenness centrality, we found that Gng13 (also known as G Protein Subunit Gamma 13, Gγ13) was localized in the core of the network, which directly regulates 103 neighboring genes (Figure 9A). To further confirm the effect of Ang II infusion on Gng13 expression, we performed qPCR analysis. Compared with that in saline control-treated LECs, the Gng13 mRNA level in Ang II-treated LECs was increased at 12 h and then decreased at 24 h (Figure 9B). Together, these findings suggest that Gng13 may play a role in the regulation of lymphangiogenesis.

## Discussion

**FIGURE 10:**
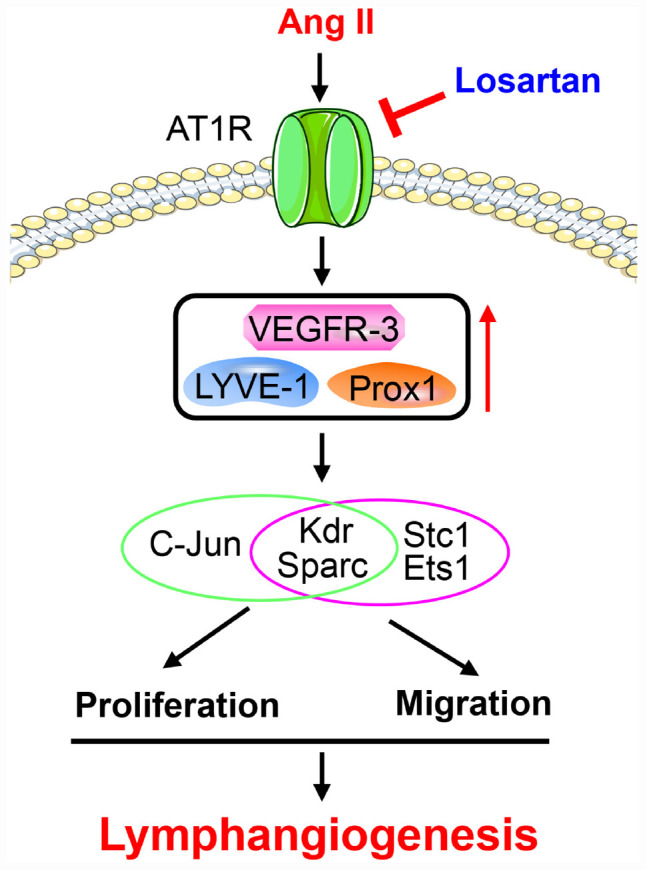
A working model for Ang II to stimulate proliferation and migration of LECs and lymphangiogenesis.

In the present study, our results for the first time showed that Ang II significantly promoted cardiac lymphangiogenesis *in vivo* and the proliferation and migration of LECs *in vitro* in a time-dependent manner. Moreover, 1,385 DEGs were identified in LECs after 12 and 24 h of Ang II treatment that are involved in multiple biological processes and signaling pathways. These genes were classified into 16 clusters based on their expression patterns and functions, and 7 clusters containing 586 genes were statistically significant. Importantly, Ang II induced the proliferation and migration and related gene expression through AT1R. Thus, the results of the present study identified new potential mechanisms by which Ang II regulates the proliferation and migration of LECs. A working model is illustrated in Figure 10.

Lymphatic remodeling is a major feature of lymphatic diseases and involves alterations in the proliferation, migration and apoptosis of LECs. Ang II is known to bind AT1R to activate these signaling pathways, which are the primary mechanisms involved in Ang II-induced inflammation, fibrosis, and oxidative stress in various tissues and cells (Dang et al., 2015; Zhang et al., 2018; Wu et al., 2019). In the present study, we extended these findings and further reveled that Ang II infusion significantly promotes cardiac lymphangiogenesis (Figure 1), but whether this effect is direct or indirect remains unclear. A previous study reported that Ang II (1 μM) treatment for 24 h had no effect on the markers CXCL10, NOS2, ICAM-1, VCAM-1, and VEGR-3, which are required for activation, adhesion and proliferation as well as the AT1R1 expression in mouse LECs, but the expression levels of other genes and proteins as well as the proliferation and migration of LECs were not analyzed (Balasubbramanian et al., 2020). However, our results showed that Ang II (500 nM) treatment significantly upregulated the mRNA and protein levels of AT1R at 12 and 24 h (Figures 4A,B) but did not affect the mRNA levels of CXCL10, NOS2, ICAM-1, and VCAM-1 at 12 or 24 h (data not shown), which are not traditional markers of LECs and are expressed by multiple cell types. Moreover, the Ang II (500 nM)-induced upregulation of lymphatic genes such as LYVE-1, Prox1, VEGF-C and VEGFR-3, and promoted the proliferation and migration of LECs (Figures 2B,C, 3A–C), which were fully inhibited by the AT1R antagonist losartan (Figures 4C–E), demonstrating that Ang II can directly promote LECs growth *in vitro*.

Increasing evidence suggests that multiple signaling pathways are crucial for lymphatic growth and function, including the VEGF-C/VEGFR-3, insulin-like growth factor-1 (IGF-1)/PI3K/Akt, MAPK2/ERK/JNK, Jak/STAT3, NF-kB, and Toll-like receptor 4 (TLR4) pathways (Flister et al., 2010; Jung et al., 2015; Hu et al., 2019). VEGF-C-VEGFR-3 signaling has been shown to be apical in developmental lymphangiogenesis and lymphatic vessel remodeling, which play central roles in maintaining the tissue fluid balance and immune response in both physiological and pathological conditions (Norrmén et al., 2011). To identify the DEGs, biological processes and signaling pathways involved in the proliferation and migration of LECs induced by Ang II, we performed time-series microarray analyses, which have been used to examine gene expression profiles in different tissues, including heart, vessel and kidney tissues after Ang II infusion (Dang et al., 2015; Zhang et al., 2018; Wu et al., 2019). We identified 1,385 DEGs that were primarily associated with multiple signaling pathways, such as including olfactory transduction, alcoholism, and RIG-I-like receptor (Figure 2), which are not previously identified. However, the roles of these signaling pathways in the proliferation and migration of LECs or lymphangigenesis remain to be explored in future studies.

To determine the profiles of DEGs in Ang II-stimulated LECs, we further analyzed the time-series microarray results and observed that that the Ang II-induced 1,385 DEGs could be classified into 16 clusters based on their expression patterns and functions, where 7 clusters (Nos. 13, 2, 8, 15, 7, 3, and 12) containing 586 genes were statistically significant (Figures 2B, 3). Furthermore, the DEGs in clusters 13, 8, 15, and 12 increased after Ang II treatment in a time dependent manner, whereas, the DEGs in clusters. 2, 7, and 3 gradually decreased over time (Figure 3B). Interestingly, several well-known lymphangiogenesis-related genes, such as LYVE-1, Prox1, and VEGFR-3 (Flt4) were significantly upregulated IECs in response to Ang II stimulation (Figures 1B–D, 2B,C). Moreover, the expression of several lymphatic proliferation- and migration-related genes, including Kdr, c-Jun, Stc1, Sparc and Ets1 were significantly altered in Ang II-treated LECs, and this effect was markedly reversed by losartan (Table 1 and Figures 7A, 8A). The homeobox transcription factor Prox1 plays pivotal roles in the development of embryonic lymphatics and in the maintenance of the adult lymphatic system by regulating various LEC related factors, such as VEGFR-3 (Yang and Oliver, 2014). Kdr (a VEGFR-2 homolog) is indispensable for craniofacial lymphangiogenesis in zebrafish and is involved in the proliferation and migration of LECs (Vogrin et al., 2019). c-Jun participates in the proliferation of LECs (Wissmann and Detmar, 2006), while Ets1, an important transcription factor of the Ets family, is required for Kaposi’s sarcoma-associated herpesvirus (KSHV)-induced expression of VEGFR-3 and lymphangiogenesis (Gutierrez et al., 2013). Moreover, NF-kB is able to function together with Prox1 to induce the expression of VEGFR-3 in LECs (Flister et al., 2010). Importantly, among the identified DEGs in Ang II-treated LECs (Figure 7B), the mRNA levels of Ace, Hist1H2Ak, Olfr77 and Olfr1195 were significantly upregulated, while the those of Dusp10, Hes1, Psmc4 and Klf9 were markedly decreased (Figure 7B). The roles of these newly identified genes in the regulation of lymphatic growth and lymphangiogenesis remain to be investigated. Taken together, these results indicate that many genes are involved in Ang II-induced proliferation and migration of LECs.

Olfactory transduction is a series of events that odor molecules interact with G-protein-coupled olfactory receptors in nose, which initiates a neuronal response through heterotrimeric G-proteins (also known as guanine nucleotide-binding proteins) consisting of α, β and γ subunits. The β and γ subunits are required for the GTPase activity and G protein-effector interaction (Anholt and Rivers, 1990). Previous studies reported that Gng13 is expressed in the olfactory epithelium, particularly in the cilia of the olfactory sensory neurons (OSNs) and glomeruli of the main olfactory bulb (Li et al., 2013; Liu et al., 2018). Moreover, Gng13 can form a functional G-protein with Gβ1 and Gαolf that is critical to mammalian olfaction (Li et al., 2013), and plays an essential role in odor-triggered social behaviors including male–male aggression (Liu et al., 2018). Interestingly, our gene co-expression network analyses revealed that Gng13 (also known as G protein subunit gamma 13, Gγ13) appears at the core of the gene network, and directly regulated 103 neighboring olfactory receptor genes (Figure 9), suggesting that Gng13 may play a critical role in Ang II-induced proliferation and migration of LECs in response to Ang II treatment (Figure 9).

The limitations of this study include: (1) The rigorous controls are required to evaluate the lymphangiogenic effects of Ang II at different time points after Ang II treatment; (2) it is unclear that the mechanism by which Ang II upregulates the expression of gng13 in LECs; (3) the role of Gng13 in the regulation of the olfactory signal transduction and lymphangiogensis after Ang II stimulation.

## Conclusion

Our results revealed, for the first time, that Ang II treatment induced changes in the expression of 1,385 genes at 12 or 24 h, which likely were associated with the proliferation and migration of LECs. These genes were primarily involved in multiple biological processes and signaling pathways, such as olfactory transduction, Jak-STAT signaling pathway, alcoholism, and RIG-I-like receptor signaling pathway. Importantly, Ang II directly promoted the proliferation and migration of LECs and lymphangiogenesis through AT1R *in vitro* and *in vivo*.

## Data Availability Statement

The microarray data has been deposited into the Gene Expression Omnibus (accession: GSE150409, https://www.ncbi.nlm.nih.gov/geo/query/acc.cgi?acc=GSE150409).

## Ethics Statement

The animal study was reviewed and approved by the Animal Care and Use Committee of Dalian Medical University, and conformed to the Guide for the Care and Use of Laboratory Animals published by the U.S. NIH. Written informed consent was obtained from the owners for the participation of their animals in this study.

## Author Contributions

H-HL conceived the project. Q-YL and JB performed *in vitro* experiments and analyzed the results. All authors contributed to the article and approved the submitted version.

## Conflict of Interest

The authors declare that the research was conducted in the absence of any commercial or financial relationships that could be construed as a potential conflict of interest.
